# An integrative radiological, histopathological and molecular analysis of pediatric pontine histone-wildtype glioma with MYCN amplification (HGG-MYCN)

**DOI:** 10.1186/s40478-019-0738-y

**Published:** 2019-06-10

**Authors:** A. Tauziède-Espariat, M-A Debily, D. Castel, J. Grill, S. Puget, M. Sabel, K. Blomgren, A. Gareton, V. Dangouloff-Ros, E. Lechapt, N. Boddaert, P. Varlet

**Affiliations:** 1 Department of Neuropathology, GHU Paris-Neurosciences, Sainte-Anne Hospital 1, rue Cabanis, 75014 Paris, France; 2 UMR8203, Vectorologie et thérapeutiques anticancéreuses, CNRS, Gustave Roussy, University of Paris-Sud, University of Paris-Saclay, 94805 Villejuif cedex, France; 3 University of Evry, Université Paris-Saclay, 91057 Evry cedex, France; 4 Departement of Pediatric Oncology, Gustave Roussy Institute, University of Paris-Sud, Universite Paris-Saclay, 94805 Villejuif, France; 5 Department of Pediatric Neurosurgery, Necker Hospital, APHP, Universite Paris Descartes, Sorbonne Paris Cite, 75015 Paris, France; 6 Department of Pediatric Oncology, Childhood Cancer Centre Queen Silvia Children’s Hospital, Sahlgrenska University hospital, University of Gothenburg, Gothenburg, Sweden; 7 Department of Women’s and Children’s Health, Karolinska Institute, Stockholm, Sweden; 8 Department of Radiology, Necker Hospital, APHP, Universite Paris Descartes, Sorbonne Paris Cite, 75015 Paris, France

The 2016 WHO Classification of tumours of the central nervous system has introduced a new histomolecular entity, the midline diffuse glioma, H3K27 M-mutant [8]. This entity represents an infiltrative high-grade glioma (HGG) (grade IV) of the pons and the brainstem, which mainly effects children. It harbors K27 M mutations of *H3F3A, HIST1H3B/C* or *HIST2H3A/C* genes. In the pons, morphological differential diagnoses are numerous with a large spectrum of tumours ranging from benign such as pilocytic astrocytoma to malignant such as embryonal tumour with multilayered rosettes (ETMR). From the reclassification of CNS-PNETs in the study by Sturm et al.*,* HGG-MYCN was described in 28/323 cases (9%), mainly located in the cerebral hemispheres. Moreover, in a recent series of pontine gliomas, three molecular subgroups were defined: H3K27 M-mutant, MYCN-amplified and silent [1]. The MYCN subgroup was the least frequent, with only 8% of cases (4/47) [1]. This corresponds to a very rare tumour and very few radiological, clinical and histopathological data are available in the literature. In our center, six cases of HGG-MYCN were diagnosed by whole exome sequencing (WES) based on the co-amplification of *MYCN* and *ID2* (2 cases from BIOMEDE cohort) [3]. Herein, our aim is to describe the clinical, imaging, histopathological, immunohistochemical and molecular features of these cases to better characterize them.

Concordant with recent literature, the median age of our patients was 3.7 (1 to 7 years old) and concerned 4 girls (Cases 2, 3, 4 and 6) and 2 boys (Cases 1 and 5) [1]. Clinically, all our patients presented similarly to midline diffuse gliomas, H3K27 M-mutant, with a short clinical history (< 6 months of symptom duration) and the presence of a pontine tumour infiltrating at least 50% of the pons. Radiologically, all tumors were centered in the pons, with variable involvement of the mesencephalon and the middle cerebellar peduncle. None was calcified nor hemorrhagic. HGG-MYCN displayed necrosis at first presentation, with annular enhancement, larger than in H3K27 M gliomas. Furthermore, diffusion was more restricted than classically reported in diffuse intrinsic pontine gliomas (*n* = 5, median Apparent Diffusion Coefficient: 570 μm^2^/s, IQR [543–734], versus 1504 μm^2^/s in Calmon et al.), and relative Cerebral Blood Flow was higher using arterial spin labeling (*n* = 4, median 2.1, IQR [1.7–2.8] versus 1.1 in Calmon et al.) Relative Cerebral Blood Volume using dynamic susceptibility contrast perfusion MRI was in the usual range (*n* = 3, values: 1.1, 1.8, 3.8) (Fig. 1) [2]. All patients died of their disease, two directly following a surgical biopsy, one (Case 3) after 1 month of chemotherapy (VP16 carboplatin) and three (Cases 4, 5 and 6) after radiotherapy and chemotherapy. The mean overall survival of our patients was 3.8 months and the median overall survival was 4.2 months, which is shorter than midline diffuse glioma, H3 K27 M-mutant [5, 6, 10].

**Fig. 1:**
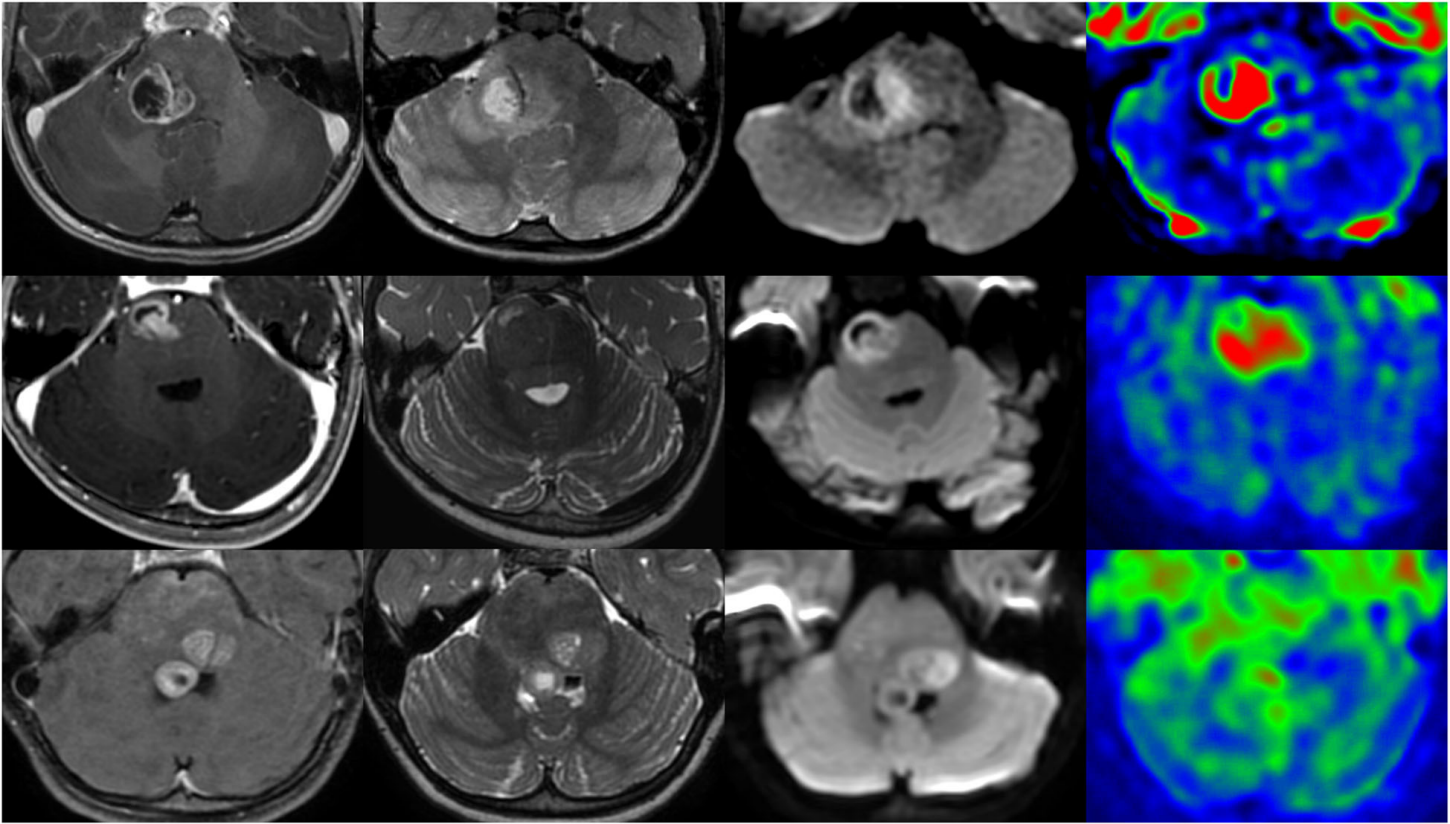
Radiological features of three pontine HGG-MYCN. Each line represents representative images of each patient (First line: Case 1; second line: Case 3; third line: Case 4). First row shows T1-weighted images after contrast media injection, second row T2-weighted images, third row diffusion-weighted images, and fourth row cerebral blood volume map using arterial spin labeling. All tumors display necrotic part with intense annular enhancement and surrounding T2-weighted hyperintense signal. Diffusion is restricted in the solid part of the tumors. Cerebral blood flow is high using arterial spin labeling

Histopathologic examination showed a well circumscribed tumor from the parenchyma with only a few cells infiltrating the surrounding parenchyma (Fig. 2a). The multinodular, undifferentiated neoplasm presented with a subtle transition between spindle cells and nodules of epithelioid cells (Fig. 2a-e). In all cases, malignancy was obvious with high mitotic count, high proliferation index (mean MIB index of 51%), necrosis and microvascular proliferation (Fig. 2 c and f). The neoplastic cells had round to oval nuclei, vesicular to coarse chromatin, prominent nucleoli and a scant to moderate amount of eosinophilic cytoplasm (Fig. 2 c and e). There was no rhabdoid component or rosettes.

**Fig. 2:**
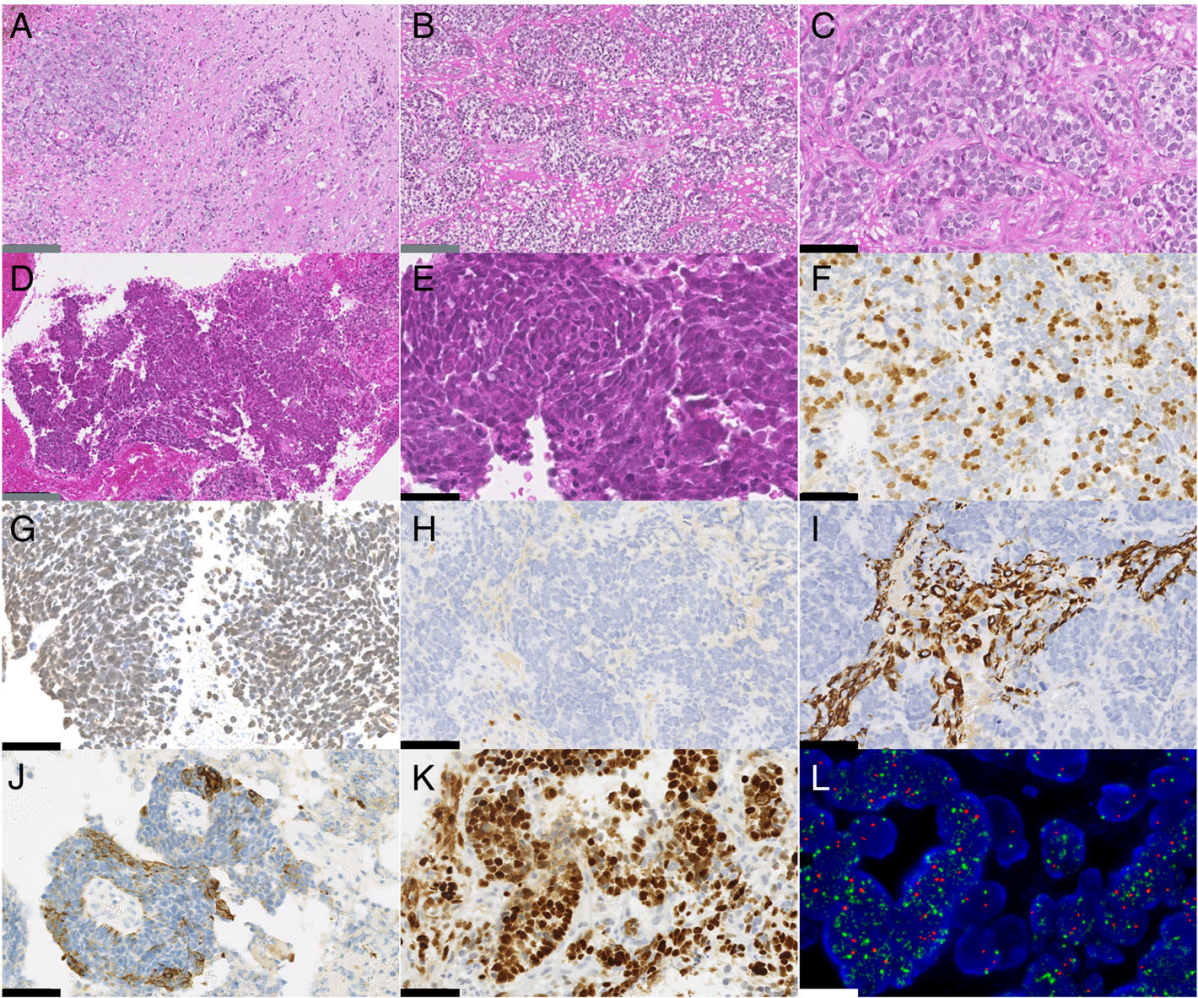
Histomolecular features of HGG-MYCN. **a** Nodules and isolated tumour cells infiltrating the pons parenchyma (Case 2, HPS, × 100 magnification). **b** Dense proliferation of tumour cells organized in nodules (Case 2, HPS, × 100 magnification). **c** Hyperchromatic undifferenciated tumour cells presenting, anisocaryotic nuclei with numerous mitoses (Case 2, HPS, × 400 magnification). **d** highly cellular and undifferenciated proliferation (Case 3, HPS, × 100 magnification). **e** Hyperchromatic tumour cells presenting anisocaryotic nuclei with numerous apoptotic bodies (Case 3, HPS, × 400 magnification). **f** Elevated index of proliferation (Case 2, MIB, × 400 magnification). **g** Preserved nuclear expression of H3K27me3 by tumour cells (Case 3, × 400 magnification). **h** Lack of expression of Olig2 in tumour cells with positive internal control (residual glial cells) (Case 2, × 400 magnification). **i** Focal expression of GFAP by tumour cells (Case 2, × 400 magnification). **j** Expression of CKAE1/AE3 in one case (Case 3, × 400 magnification). **k** Nuclear accumulation of p53 (Case 2, × 400 magnification). **l** High-level of MYCN amplification by FISH analysis (Case 4, MYCN locus in green signals and control centromeric in red signals). Black scale bars represent 50 μm, grey scale bars represent 100 μm and the white scale bar represent 5 μm

All cases of pontine HGG-MYCN did not express the H3K27 M mutated protein and presented a preserved expression of H3K27me3 (Fig. 2 g). In two cases (Cases 1 and 2), all other stains (GFAP, Olig2 – Fig. 2 h -, NFP70, synaptophysin, chromogranin A, NeuN, CD34, BRAFV600E, BCOR, NUT, Actin, Desmin, HMB45, Lin28A, EMA, CKAE1/AE3 and IDH1R132H) were negative. The four remaining cases expressed glial (Olig2 and GFAP) (Fig. 2i) and neuronal (NeuN and NFP70) markers (data not shown). All cases expressed CD56 and Vimentin. Moreover, case 3 presented a pluriphenotypic pattern with focal expression of epithelial marker (CKAE1/AE3) (Fig. 2j). In addition, the expression of INI1, BRG1 and ATRX were retained in all cases. Nuclear accumulation of p53 was present in 4/5 tested cases (Cases 2, 3, 5 and 6) (Fig. 2k) which was correlated with additional *TP53* mutations found by WES. EGFR overexpression (Cases 3 and 6) and loss of PTEN (Cases 2 and 5) were mutually exclusive. No mutations of the *hTERT* promoter, *IDH1*, *IDH2*, *H3F3A*, *HIST1H3B* and *BRAF* were observed. The amplification of the *MYCN* gene was confirmed by FISH analysis Zyto*Light*® SPEC MYCN/2q11 Dual Color Probe (Zytovision, Germany) in all cases (Fig. 2l). It should be noted that *MYCN* amplification is not a specific alteration of the HGG-MYCN entity. Indeed, it may be encountered in a small portion of pediatric glioblastoma-RTKI (Receptor of Tyrosine Kinase I) and –RTKII (Receptor of Tyrosine Kinase II) [6]. However, WES analysis revealed a co-amplification of the *ID2* gene in all our cases which is not described in other types of glioblastomas. This co-amplification was described in the supra-tentorial and the pontine location of HGG-MYCN [1, 9]. *ID2* encodes the protein ID2 (Inhibitor of DNA-binding 2) which is expressed by oligodendroglial precursor cells, a progenitor cell type implicated in the oncogenesis of gliomas [4, 7, 11]. Furthermore, a subset of diffuse midline gliomas with H3.3-K27 M mutations overexpress *ID2* without amplification of this gene [1]. These data suggest a potential common *ID2*-related mechanism in pontine high-grade gliomas tumorigenesis.

We here extend the knowledge about the rare pontine HGG-MYCN representing a differential diagnosis of DIPG in young children. This tumoral entity presents with clinicoradiological and phenotypical features that clearly distinguishes it from classical midline diffuse glioma, H3K27 M-mutant and might allow for the establishment of targeted molecular therapy in the future. In small biopsy of pontine tumors, the emergence of this HGG-MYCN subgroup reinforces the necessity for valuable immunohistochemistry and FISH analyses if molecular analysis are not feasible or non-contributory. Therefore, we strongly recommend adding *MYCN* FISH analysis in the diagnostic immunohistochemical/molecular panel of pediatric brainstem tumours, similarly to H3K27M, INI1/BRG1 and Lin28A antibodies for the differential diagnoses of DIPG, AT/RT and ETMR, respectively.

## References

[1] Buczkowicz P, Hoeman C, Rakopoulos P, Pajovic S, Letourneau L, Dzamba M, Morrison A, Lewis P, Bouffet E, Bartels U, Zuccaro J, Agnihotri S, Ryall S, Barszczyk M, Chornenkyy Y, Bourgey M, Bourque G, Montpetit A, Cordero F, Castelo-Branco P, Mangerel J, Tabori U, Ho KC, Huang A, Taylor KR, Mackay A, Bendel AE, Nazarian J, Fangusaro JR, Karajannis MA, Zagzag D, Foreman NK, Donson A, Hegert JV, Smith A, Chan J, Lafay-Cousin L, Dunn S, Hukin J, Dunham C, Scheinemann K, Michaud J, Zelcer S, Ramsay D, Cain J, Brennan C, Souweidane MM, Jones C, Allis CD, Brudno M, Becher O, Hawkins C (2014). Genomic analysis of diffuse intrinsic pontine gliomas identifies three molecular subgroups and recurrent activating ACVR1 mutations. Nat Genet.

[2] Calmon R, Puget S, Varlet P, Beccaria K, Blauwblomme T, Grevent D, Sainte-Rose C, Castel D, Dufour C, Dhermain F, Bolle S, Saitovitch A, Zilbovicius M, Brunelle F, Grill J, Boddaert N (2017). Multimodal magnetic resonance imaging of treatment-induced changes to diffuse infiltrating pontine gliomas in children and correlation to patient progression-free survival. Int J Radiat Oncol Biol Phys.

[3] Castel D, Philippe C, Kergrohen T, Sill M, Merlevede J, Barret E, Puget S, Sainte-Rose C, Kramm CM, Jones C, Varlet P, Pfister SM, Grill J, Jones DTW, Debily M-A (2018). Transcriptomic and epigenetic profiling of “diffuse midline gliomas, H3 K27M-mutant” discriminate two subgroups based on the type of histone H3 mutated and not supratentorial or infratentorial location. Acta Neuropathol Commun.

[4] Havrda MC, Paolella BR, Ran C, Jering KS, Wray CM, Sullivan JM, Nailor A, Hitoshi Y, Israel MA (2014). Id2 mediates oligodendrocyte precursor cell maturation arrest and is tumorigenic in a PDGF-rich microenvironment. Cancer Res.

[5] Khuong-Quang D-A, Buczkowicz P, Rakopoulos P, Liu X-Y, Fontebasso AM, Bouffet E, Bartels U, Albrecht S, Schwartzentruber J, Letourneau L, Bourgey M, Bourque G, Montpetit A, Bourret G, Lepage P, Fleming A, Lichter P, Kool M, von Deimling A, Sturm D, Korshunov A, Faury D, Jones DT, Majewski J, Pfister SM, Jabado N, Hawkins C (2012). K27M mutation in histone H3.3 defines clinically and biologically distinct subgroups of pediatric diffuse intrinsic pontine gliomas. Acta Neuropathol (Berl).

[6] Korshunov Andrey, Schrimpf Daniel, Ryzhova Marina, Sturm Dominik, Chavez Lukas, Hovestadt Volker, Sharma Tanvi, Habel Antje, Burford Anna, Jones Chris, Zheludkova Olga, Kumirova Ella, Kramm Christof M., Golanov Andrey, Capper David, von Deimling Andreas, Pfister Stefan M., Jones David T. W. (2017). H3-/IDH-wild type pediatric glioblastoma is comprised of molecularly and prognostically distinct subtypes with associated oncogenic drivers. Acta Neuropathologica.

[7] Liu C, Sage JC, Miller MR, Verhaak RGW, Hippenmeyer S, Vogel H, Foreman O, Bronson RT, Nishiyama A, Luo L, Zong H (2011). Mosaic analysis with double markers reveals tumor cell of origin in glioma. Cell.

[8] Louis DN, Ohgaki H., Wiestler O. D., Cavenee WK, Ellison DW, Figarella-Branger D, Perry A, Reifenberger G, von Deimling A. The 2016 World Health Organization classification of tumors of the central nervous system, 4th ed. 2016. 10.1007/s00401-016-1545-1 27157931

[9] Sturm D, Orr BA, Toprak UH, Hovestadt V, Jones DTW, Capper D, Sill M, Buchhalter I, Northcott PA, Leis I, Ryzhova M, Koelsche C, Pfaff E, Allen SJ, Balasubramanian G, Worst BC, Pajtler KW, Brabetz S, Johann PD, Sahm F, Reimand J, Mackay A, Carvalho DM, Remke M, Phillips JJ, Perry A, Cowdrey C, Drissi R, Fouladi M, Giangaspero F, Łastowska M, Grajkowska W, Scheurlen W, Pietsch T, Hagel C, Gojo J, Lötsch D, Berger W, Slavc I, Haberler C, Jouvet A, Holm S, Hofer S, Prinz M, Keohane C, Fried I, Mawrin C, Scheie D, Mobley BC, Schniederjan MJ, Santi M, Buccoliero AM, Dahiya S, Kramm CM, von Bueren AO, von Hoff K, Rutkowski S, Herold-Mende C, Frühwald MC, Milde T, Hasselblatt M, Wesseling P, Rößler J, Schüller U, Ebinger M, Schittenhelm J, Frank S, Grobholz R, Vajtai I, Hans V, Schneppenheim R, Zitterbart K, Collins VP, Aronica E, Varlet P, Puget S, Dufour C, Grill J, Figarella-Branger D, Wolter M, Schuhmann MU, Shalaby T, Grotzer M, van Meter T, Monoranu C-M, Felsberg J, Reifenberger G, Snuderl M, Forrester LA, Koster J, Versteeg R, Volckmann R, van Sluis P, Wolf S, Mikkelsen T, Gajjar A, Aldape K, Moore AS, Taylor MD, Jones C, Jabado N, Karajannis MA, Eils R, Schlesner M, Lichter P, von Deimling A, Pfister SM, Ellison DW, Korshunov A, Kool M (2016). New brain tumor entities emerge from molecular classification of CNS-PNETs. Cell.

[10] Sturm D, Witt H, Hovestadt V, Khuong-Quang D-A, Jones DTW, Konermann C, Pfaff E, Tönjes M, Sill M, Bender S, Kool M, Zapatka M, Becker N, Zucknick M, Hielscher T, Liu X-Y, Fontebasso AM, Ryzhova M, Albrecht S, Jacob K, Wolter M, Ebinger M, Schuhmann MU, van Meter T, Frühwald MC, Hauch H, Pekrun A, Radlwimmer B, Niehues T, von Komorowski G, Dürken M, Kulozik AE, Madden J, Donson A, Foreman NK, Drissi R, Fouladi M, Scheurlen W, von Deimling A, Monoranu C, Roggendorf W, Herold-Mende C, Unterberg A, Kramm CM, Felsberg J, Hartmann C, Wiestler B, Wick W, Milde T, Witt O, Lindroth AM, Schwartzentruber J, Faury D, Fleming A, Zakrzewska M, Liberski PP, Zakrzewski K, Hauser P, Garami M, Klekner A, Bognar L, Morrissy S, Cavalli F, Taylor MD, van Sluis P, Koster J, Versteeg R, Volckmann R, Mikkelsen T, Aldape K, Reifenberger G, Collins VP, Majewski J, Korshunov A, Lichter P, Plass C, Jabado N, Pfister SM (2012). Hotspot mutations in H3F3A and IDH1 define distinct epigenetic and biological subgroups of glioblastoma. Cancer Cell.

[11] Wu Y, Liu Y, Levine EM, Rao MS (2003). Hes1 but not Hes5 regulates an astrocyte versus oligodendrocyte fate choice in glial restricted precursors. Dev Dyn Off Publ Am Assoc Anat.

